# Challenging presentations of cavernous sinus thrombophlebitis

**DOI:** 10.1007/s12348-011-0053-7

**Published:** 2011-12-04

**Authors:** Courtney L. Kraus, Susan M. Culican

**Affiliations:** Department of Ophthalmology and Visual Sciences, Washington University School of Medicine, 660 South Euclid Ave, St. Louis, MO 63110 USA

**Keywords:** Cavernous sinus thrombosis, Septic thrombophlebitis, Cranial nerve palsies, Imaging

## Abstract

**Aim:**

The purpose of this study was to describe two challenging cases of septic cavernous sinus thrombosis (CST), which presented with vastly different clinical signs and symptoms.

**Methods:**

We present two cases of CST with markedly differing clinical presentations, medical comorbidities, and degree of impairment. Initial imaging of each patient failed to show thromboembolic disease.

**Results:**

Both patients required multiple imaging procedures to arrive at the correct diagnosis. Each child did respond to treatment once the correct diagnosis was made.

**Conclusion:**

CST can have a highly variable clinical presentation, from a subtle sixth nerve palsy to complete ophthalmoplegia and loss of periorbital sensation and corneal reflex. Onset of symptoms may be acute and fulminant or indolent and delayed. The diagnosis is challenging, requiring clinical suspicion and confirmation by imaging. These cases illustrate the importance of retaining clinical suspicion when cranial nerve palsies persist and how valuable rescanning a patient can be.

## Introduction

Cavernous sinus thrombosis (CST) is a rare phenomenon that requires clinical suspicion and emergent imaging for correct diagnosis. We report two cases of CST where initial imaging failed to show a thrombus. When both patients continued to deteriorate despite medical and surgical intervention, repeat scanning confirmed suppurative thromboembolism.

## Case 1

A 16-year-old girl presented to the emergency department with 3 days of headache, diplopia and progressive right-sided periorbital edema, and ptosis. Her medical history was significant for type 1 diabetes mellitus and asthma, for which she took oral prednisone.

On initial examination, visual acuity was decreased to 20/40 in her right eye. Right pupil was fixed and dilated with an afferent pupillary defect. External exam demonstrated significant upper and lower lid edema and erythema, ptosis, and moderate proptosis. She had diminished sensation in the distribution of cranial nerve (CN) V1. The right eye was deviated down and out with complete impairment of elevation, adduction, and depression—consistent with a CN III palsy. The remainder of the eye examination was normal. Examination of the oropharynx was unremarkable.

**Fig. 1:**
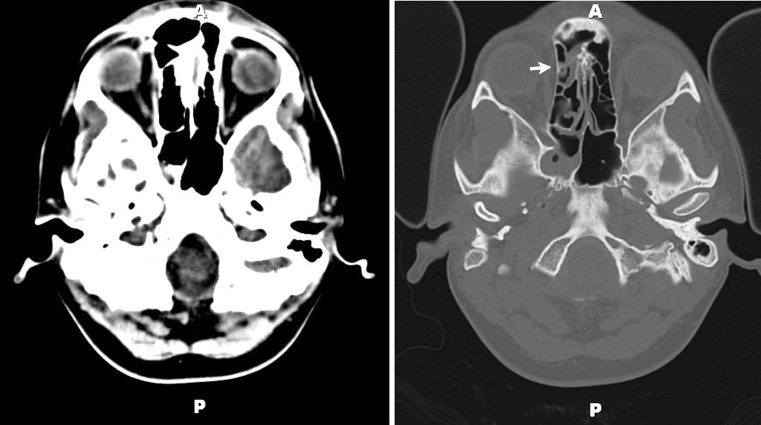
Non-contrast CT head. **a** An axial image showing mucosal thickening within the right sphenoid and ethmoid sinuses. Bone window from the same study more clearly demonstrating sinus disease (*white arrows*)

Head computed tomography (CT) showed sinusitis (Fig. 1), which was presumed to have caused an orbital cellulitis and the resulting nerve palsies. Broad spectrum antibiotic coverage was initiated. The following day, brain and brain stem magnetic resonance imaging (MRI) was unimpressive, demonstrating normal enhancement of the cavernous sinus without evidence of thrombosis. MR angiography (MRA) and venography (MRV) were also within normal limits. Antifungals were added to cover mucormycosis. Steroids were discussed, but deferred given the concern for infection.

Forty eight hours of antibiotics and antifungals improved the preseptal cellulitis. However, vision and motility worsened with loss of abduction of the right eye. Fundoscopic exam revealed a pale optic disk. Suspicion of mucormycosis led to sinus exploration by otolaryngology (ENT) which revealed no purulent material, erosive disease or evidence of mucor.

**Fig. 2:**
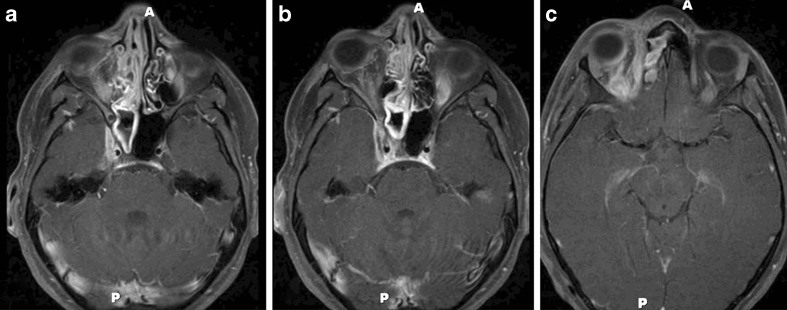
MRI of head and orbits performed with and without contrast. Serial non-contrast axial images showing worsening right orbital cellulitis and enlargement of the right cavernous sinus (**a**–**c**)

Despite days of antibiotics and antifungals, the patient's course had deteriorated. Now faced with impairment of CN II, III, V1, and VI, the decision was made to reimage. MRI was repeated and revealed right CST (Fig. 2). This necessitated initiation of steroids and anticoagulation. Four days later, her vision improved to counting fingers. After 5 weeks of intravenous antibiotic and antifungal therapy, a steroid taper and 6 months of anticoagulation, her vision stabilized at 20/60.

## Case 2

A 19-year-old girl presented to an outside hospital with complaints of malaise, fever, and headache. A positive rapid monospot test leads to a diagnosis of mononucleosis and conservative management was recommended. Over the next 72 h, her headache worsened and she developed nausea, emesis, ptosis, and diplopia. She re-presented to the hospital; at this time, blood cultures were drawn. Cultures were positive for *Fusobacterium necrophorum*, *Arcanobacterium hemolyticum*, and group C beta hemolytic streptococcus. She was transferred to our hospital.

At presentation she had significant left orbital pain, proptosis, and ptosis. She reported blurred vision in the left eye; however, visual acuity was 20/20. She had impairment of left eye abduction consistent with a CN VI palsy. No other cranial nerve palsies were detected. External exam was notable for minimal left-sided ptosis; the remainder of slit lamp exam was normal. MRI at that time showed extensive paranasal sinus disease without extension into the orbit. Broad spectrum antibiotic coverage was initiated.

**Fig. 3:**
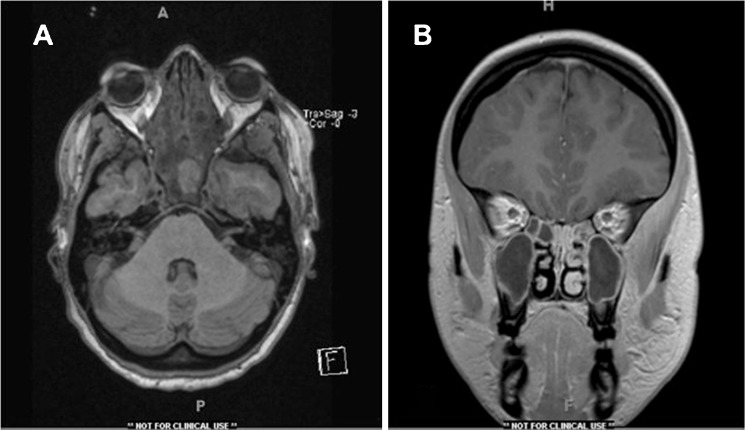
MRI of head and orbits, performed with and without contrast. **a** Axial view depicting extensive sinusitis. **b** Coronal view showing thickening within the cavernous sinus with masticator space abscess

ENT took the patient for sinus exploration, which revealed purulent material within the maxillary, posterior ethmoid, and sphenoid sinuses. Cultures of this material were again positive for the previously isolated *F. necrophorum*, *A. hemolyticum*, and group C beta hemolytic streptococcus. The following day blood cultures were repeated and found to be negative for bacteria or fungus. Despite antibiotic therapy, the patient remained febrile and CN VI palsy persisted. Concern for the involvement of the cavernous sinus led to repeat imaging.

MRI revealed thickening of the cavernous sinus consistent with CST (Fig. 3). Interval development of a left hypothalamic ischemic infarct and a left masticator space abscess was also noted. Management at this point included drainage of the abscess and continued antibiotic therapy. Extensive discussion was given to whether anticoagulation was indicated in the setting of cavernous sinus thrombosis; however, initiation was deferred. At this point, two MRVs and a jugular venosonogram had failed to show thrombophlebitis of the internal jugular vein.

The following day, the patient developed a right-sided CN IX palsy. MRI was repeated and a thrombosis of the internal jugular vein was discovered. At this point, the decision was made to start anticoagulation. Thereafter, she demonstrated rapid resolution of her fever and cessation of further thromboembolic events. She completed 6 weeks of antibiotics and anticoagulation. At the time of follow-up, she continued to demonstrate a mild ptosis and abduction defect.

## Discussion

The cavernous sinus, with its central location and many direct and indirect vascular connections, is particularly vulnerable to septic thrombosis from the face, nose, tonsils, teeth, and ears. A valveless system of sinuses and veins allows for easy spread of bacteria. Bacterial infection of the orbital structures is more frequent in pediatric patients, secondary to the increased frequency of upper respiratory tract and paranasal infections [1]. The most frequently encountered organisms are *Staphylococcus aureus* (35%), *Streptococcus pneumoniae* and other streptococci species, Gram-negative bacilli, and anaerobes [2–4]. Other infections, such as mucormycosis and aspergillosis, are of greater concern in diabetics, the immunocompromised, and other high-risk patient populations.

The source of infection in our first patient was likely sinusitis. The second patient's bacteremia placed her at risk of septic thrombophlebitis. Initial imaging in both girls showed inflammation of the paranasal sinuses, a common source of bacteria. The oropharynx is another source of septic thrombi. *F. necrophorum*, a commensual anaerobe found in the oropharynx, is responsible for the thrombophlebitis seen in Lemierre's syndrome and has caused rare cases of CST [5]. This organism was identified on our second patient's blood cultures, along with the more commonly implicated streptococcus species and likely contributed to her multiple thromboembolic events. Mastoiditis is an increasingly rare infectious source for CST due to widespread adoption of the 7-valent pneumococcal conjugate vaccine [6].

After a comprehensive history and physical exam, neuroimaging is one of the most useful tools for evaluating the orbits. High-resolution MRI is the modality of choice in CST. It can detect all stages of thrombus formation, whereas CT can be inconclusive secondary to bone artifact [7]. Filling defects within the cavernous sinus and expansion of tributary veins and venous sinuses are seen in cases of CST. However initial scans can be negative, so evaluating physicians must be diligent in cases where the suspicion is high for a vascular obstruction. Venous dilation can also be seen in carotid-cavernous fistulas, Graves orbitopathy, orbital pseudotumor, and meningiomas. Therefore, CT angiography or MRA may also be used to identify the cause of presenting symptoms [1, 8].

The prognosis and clinical course of CST have been dramatically changed by prompt recognition and treatment with antibiotics [9]. Intravenous corticosteroids have been theorized to rein in the prothrombotic state seen in orbital infections. They are first-line therapy for cases of idiopathic orbital inflammation, where they have proven efficacy in reducing perivascular inflammation and improving cranial nerve dysfunction [10]. However, their use in conjunction with antibiotics has not been shown to improve outcomes in orbital cellulitis [11, 12]. Their role in CST is not known. The role of anticoagulation for septic thromboemboli has not been examined in randomized controlled trials. However, heparin administration in the early period of hospitalization has been associated with a reduction in diplopia from cranial nerve dysfunction and blindness secondary to optic nerve damage [13, 14]. Four to 6 weeks of anticoagulation with warfarin is recommended after initial heparin therapy.

We presented two cases of patients ultimately diagnosed with septic thromboembolic disease. After presenting with one or more cranial nerve palsies and with imaging that revealed sinusitis, antibiotic therapy failed to improve their courses. Despite the concerted efforts of a multidisciplinary team comprising ENT, infectious disease, critical care, and ophthalmology, both girls worsened. CST is thought to be a rare diagnosis with today's widespread use of antibiotics for oropharyngeal infections. However, a low threshold for repeatedly considering septic thromboembolism was vitally important in the decision to rescan each girl and should be regarded as a crucial and astute step leading to their recovery. Every subspecialist who encounters a patient with cranial nerve palsies and sinusitis must consider CST and maintain a low threshold for rescanning.

## Abbreviations

CST: Cavernous sinus thrombosis
CT: Computed tomography
CN: Cranial nerve
MRA: Magnetic resonance angiography
MRI: Magnetic resonance imaging
MRV: Magnetic resonance venography
ENT: Otolaryngology
